# Use of Topical Antibiotics in Open Fractures: A Systematic Review and Meta-Analysis

**DOI:** 10.7759/cureus.92261

**Published:** 2025-09-14

**Authors:** Gayle Caruana, Ryan Giordmaina

**Affiliations:** 1 Surgery, Mater Dei Hospital, Msida, MLT; 2 Orthopaedics and Traumatology, Mater Dei Hospital, Msida, MLT

**Keywords:** antibiotic usage, open fracture, surgical site infection, topical antibiotics, trauma

## Abstract

Open fractures are associated with a high risk of surgical site infection (SSI) due to impaired vascularity, extensive soft tissue injury, and contamination. Despite prompt systemic antibiotic administration being the standard of care, infection rates remain substantial. The adjunctive use of topical antibiotics has been proposed to further reduce this risk.

A systematic review and meta-analysis was conducted in accordance with the Preferred Reporting Items for Systematic Reviews and Meta-Analyses (PRISMA) guidelines. PubMed, Scopus, and the Cochrane Library databases were searched for studies published between 2015 and 2025. Six studies (one randomized controlled trial and five cohort studies) involving 2,982 patients were included, of whom 758 received adjunctive topical antibiotics. The pooled analysis demonstrated a significantly lower SSI rate in patients receiving both systemic and topical antibiotics compared with systemic antibiotics alone (7.26% vs. 10.12%; p=0.0197). No significant difference was observed between monotherapy with vancomycin and dual therapy with vancomycin plus tobramycin (p=0.0602). No increase in systemic adverse events was reported, although one study noted a higher incidence of wound-healing complications with vancomycin powder.

Adjunctive topical antibiotic administration was associated with a statistically significant reduction in SSI rates following open fractures. However, heterogeneity in study design, outcome definitions, and interventions limits the strength and generalizability of the evidence. Large, high-quality randomized trials are needed to establish optimal regimens, evaluate long-term safety, and determine cost-effectiveness.

## Introduction and background

Open fractures, defined as fractures in which the bone communicates with the external environment through a soft tissue wound, are frequently associated with extensive soft tissue damage and bone disruption. These injuries carry a substantial risk of infection, leading to increased morbidity, prolonged hospitalization, and a considerable financial burden on healthcare systems. Historically, management has centred on urgent surgical debridement, stabilization of the fracture, and the early administration of systemic antibiotic prophylaxis. While these measures have significantly reduced mortality and infection compared with pre-antibiotic eras, infection rates in open fractures remain high, ranging from 2% to 38% [1]. The risk is largely influenced by fracture complexity, the degree of wound contamination, and the extent of soft tissue compromise, underscoring the need for additional preventive strategies.

Given that systemic antibiotics may not achieve adequate tissue concentrations in areas of compromised vascularity, attention has turned toward topical antibiotic therapies. These include the direct application of powders such as vancomycin and tobramycin at the surgical site. Evidence from other fields, particularly spinal surgery, has demonstrated promising reductions in surgical site infection (SSI) with topical antibiotic use [2,3]. Building on this, newer delivery systems, such as antibiotic-containing pastes and antibiotic-impregnated bone substitutes, have emerged as adjunctive options in orthopaedic trauma care.

Despite increasing interest, the clinical effectiveness of topical antibiotics in open fractures remains uncertain. Some studies suggest a protective effect, whereas others show minimal or no benefit. These inconsistencies likely reflect variability in study design, patient populations, antibiotic type and dosing, and outcome definitions. As a result, consensus guidelines have yet to endorse topical antibiotic use as standard practice.

This systematic review and meta-analysis aims to critically evaluate the available evidence on topical antibiotic therapies in open fractures, with the objective of clarifying their clinical utility and informing both surgical practice and future research.

## Review

Methodology

This systematic review was prospectively registered with the International Prospective Register of Systematic Reviews (PROSPERO) with registration number CRD420251121084. The protocol was developed prior to data extraction and followed throughout the study. Conducted in accordance with the Preferred Reporting Items for Systematic Reviews and Meta-Analyses (PRISMA) 2020 guidelines, this systematic review and meta-analysis aimed to evaluate whether the adjunctive use of topical antibiotics, in addition to systematic prophylaxis, reduces the incidence of SSI in adult patients with open fractures. 

Eligibility Criteria

Studies were eligible for inclusion if they met the following criteria: the population consisted of adult patients aged 18-85 years with open upper or lower limb fractures classified according to the Gustilo-Anderson system (types I to III). The intervention involved the use of topical antibiotics, as either monotherapy or dual therapy, in addition to standard systemic antibiotic prophylaxis. The control group received systemic antibiotic prophylaxis alone. The primary outcome assessed was the incidence of SSI, while secondary outcomes included antimicrobial resistance, adverse events, and cost-effectiveness. Eligible study designs included randomized controlled trials (RCTs), cohort studies, case-control studies, and cross-sectional studies. Only peer-reviewed articles published in English between January 2015 and January 2025 were considered.

The rationale for this timeline was based on the increased adoption of topical antibiotic techniques following the 2015 publication of key infection-preventing guidelines in orthopaedic trauma and to capture the most recent evidence available up to the time of review.

Exclusion criteria included closed fractures, pelvic trauma, case reports, editorials, non-peer-reviewed studies, and studies involving paediatric or pregnant populations.

Types of Outcome Measures

Primary outcomes: The primary outcome of this review was to determine whether the application of topical antibiotics in addition to systemic antibiotics reduces the incidence of SSIs and lowers the rate of deep fracture-related infection following open fracture treatment.

Secondary outcomes: Secondary outcomes include evaluating the potential development of antimicrobial resistance associated with prophylactic intrawound antibiotic use, as well as examining the impact of topical antibiotic application on fracture healing, specifically the incidence of non-union. Furthermore, this review aimed to assess the cost-effectiveness and overall patient outcomes (topical and systemic) following topical antibiotic administration in open fracture surgeries.

Information Sources and Search Strategy

A comprehensive search was conducted in PubMed, Scopus, and the Cochrane Library in February 2025. Search terms included a combination of keywords "open fracture", "topical antibiotics", "local antibiotics", "intra-wound antibiotics", "trauma", "open fracture", "vancomycin", "tobramycin", and "bone infection" and Medical Subject Headings (MeSH) terms: Anti-Bacterial Agents (MeSH ID: D000900), Surgical Wound Infection (MeSH ID: D013293), Fractures, Open (MeSH ID: D050723), Wound Healing (MeSH ID: D014947), and Antimicrobial Resistance (MeSH ID: D000900 + subheading "drug resistance").

Search filters were applied for publication year (2015-2025) and human studies. References from included articles were manually screened to identify additional relevant studies. Grey literature sources such as conference abstracts and dissertations were not included in this review, which is acknowledged as a limitation.

The full search strategy for each database is provided in the Appendices.

Study Selection

Following de-duplication, two independent reviewers screened titles and abstracts against the eligibility criteria. Full tests of potentially eligible articles were then reviewed. Discrepancies between reviewers were resolved through consensus discussion.

**Figure 1:**
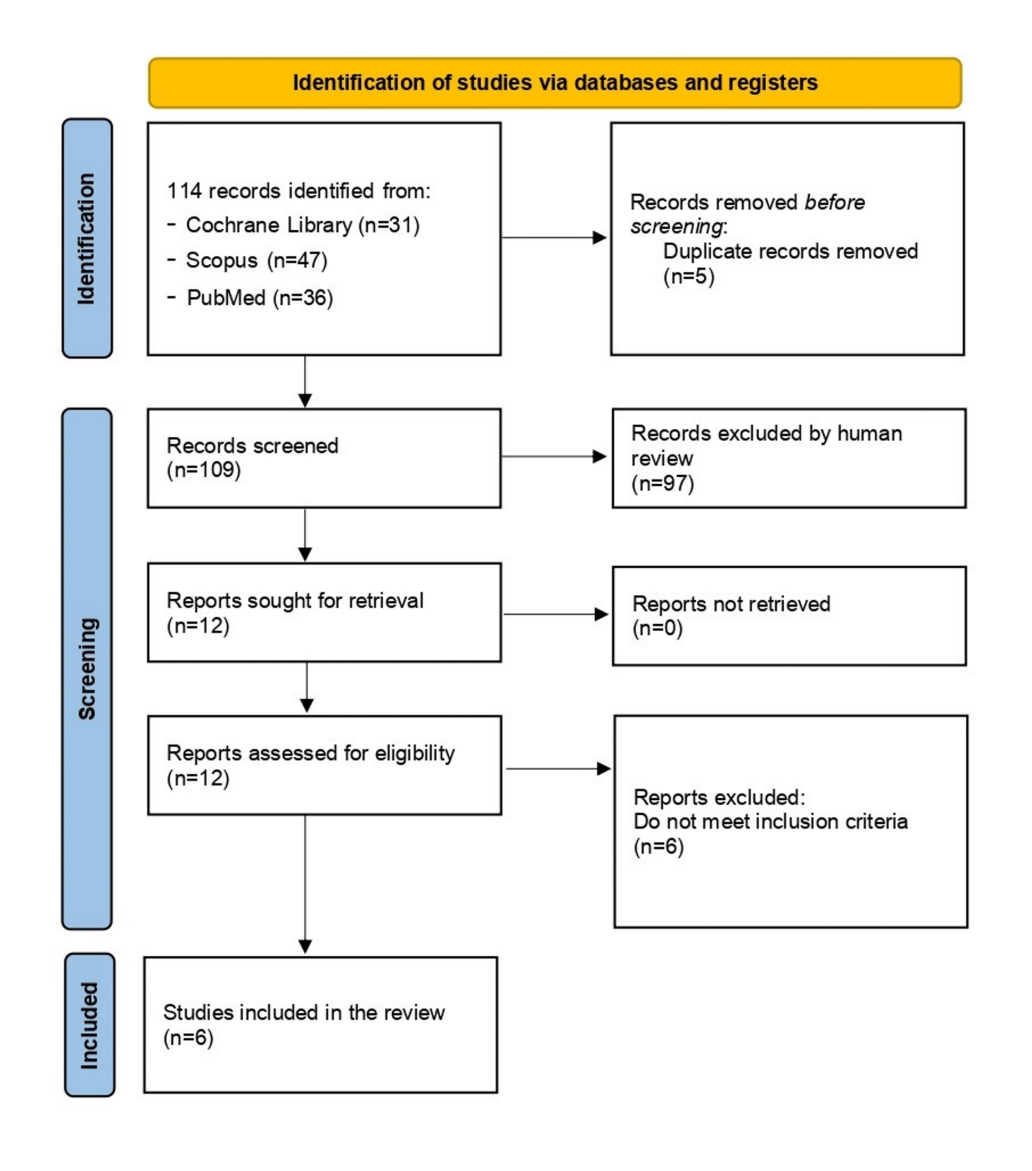
PRISMA 2020 flowchart PRISMA: Preferred Reporting Items for Systematic Reviews and Meta-Analyses

The PRISMA 2020 flowchart (Figure 1) details the selection process [4].

Data Extraction

Data were extracted independently and in duplicate using a pre-designed data extraction form in Microsoft Excel (Microsoft Corporation, Redmond, Washington, United States). Extracted variables included study characteristics, population demographics, intervention details, definition and incidence of SSI, adverse events, antibiotic resistance findings, and cost-related outcomes. Any disagreements were resolved through discussion.

Risk of Bias Assessment

The risk of bias for the included studies was assessed using tools appropriate to their study design. For the five observational studies, the ROBINS-I tool (Table 1) was used to assess bias across domains aligned with intervention-based research. The RoB 2 tool (Table 2) was applied to the single RCT, as recommended by Cochrane for evaluating randomized evidence. These tools allowed for structured, consistent, and transparent evaluation of methodological quality across different study types [5-10].

**Table 1:** Non-randomized studies of intervention 1 (ROBINS-1) traffic lights plot

	Balabanova et al. (2021) [5]	Qadir et al. (2021) [7]	Vaida et al. (2020) [8]	Pesante and Parry (2024) [9]	Taylor et al. (2024) [10]
Confounding	High	High	High	Moderate	Moderate
Selection of participants	Moderate	Moderate	Moderate	Low	Low
Classification of interventions	Moderate	Low	Low	Low	Low
Deviations from intended intervention	Moderate	Low	Moderate	Low	Low
Missing data	Moderate	Moderate	Moderate	Moderate	Moderate
Measurement of outcomes	Moderate	Low	Low	Low	Low
Selection of the reported result	Moderate	Moderate	Moderate	Moderate	Moderate
Overall risk of bias	High	High	High	Moderate	Moderate

**Table 2:** Risk of bias 2 (ROBS-2) traffic lights plot

	O'Toole et al. (2021) [6]
Randomization process	Low
Deviations from intended intervention	Low
Missing data	Moderate
Measurement of outcomes	Low
Selection of the reported result	Moderate
Overall risk of bias	Moderate

In all included studies, cefazolin was administered as the standard systemic antibiotic. For patients with cephalosporin allergy, clindamycin was used as an alternative.

Closed fractures were excluded from this review; therefore, any studies that included closed fractures within their cohorts were removed from the analysis.

Ethics Statement

As this study was based solely on previously published literature and did not involve primary data collection, no ethics approval was required.

Results

Description of the Studies

A total of 114 records were identified through database searching (31 in The Cochrane Library, 47 in Scopus, and 36 in PubMed). After the removal of five duplicates, 109 studies remained for screening.

Two independent reviewers screened titles and abstracts. After the initial screening, 12 full-text articles were assessed for eligibility. Of these, six studies met the inclusion criteria and were included in the final analysis. Reasons for exclusion included non-comparative design, inclusion of closed fractures, or lack of relevant outcome data.

Although manual reference checks were performed, no additional studies were identified. Grey literature was not systematically searched, which may have excluded relevant but unpublished data. This limitation is noted in the Discussion section.

Overview of the Selected Studies

The six included studies comprised one RCT, one prospective cohort study, three retrospective comparative cohort studies, and one retrospective case-control study. All were conducted in the United States between 2015 and 2025, involving a total of 2,982. Among these, 758 patients received topical antibiotics in addition to systemic prophylaxis.

Topical antibiotic regimens included either vancomycin alone (n=562) or vancomycin combined with tobramycin (n=196). Systemic prophylaxis protocols were consistent across studies, with intravenous cefazolin as the first-line agent or clindamycin in cases with cephalosporin.

All studies reported SSI as the primary outcome. However, definitions of SSI varied slightly, with most studies relying on the Centers for Disease Control and Prevention (CDC) criteria or clinical diagnosis requiring return to theatre. Study-level variation in follow-up duration, antibiotic administration timing (e.g., intraoperative vs. preoperative), and outcome adjudication methods were observed.

A consolidated landscape-oriented table summarizing the characteristics and outcomes of all included studies is provided in Table 3.

**Table 3:** Summary of findings SSI: surgical site infection; AKI: acute kidney injury; CDC: Centers for Disease Control and Prevention; PMMA: poly(methyl methacrylate); ED: emergency department; FRI: fracture-related infection

Study	Findings
Balabanova et al. (2021) [5]	
Title:	Incidence of Surgical Site Infection and Acute Kidney Injuries after Topical Antibiotic Powder Application.
Objective:	To compare deep SSI and AKI incidence with or without topical antibiotics
Setting:	Denver Health Level 1 Trauma Centre, United States
Level of evidence:	Low
Design:	Retrospective comparative cohort
Selection of participants:	Consecutive
Follow-up interval:	6 months
Control group:	Systemic antibiotics
Intervention group:	Vancomycin and tobramycin powder
Primary outcome:	Deep SSI, AKI
Definition of infection:	Infection requiring debridement
O'Toole et al. (2021) [6]	
Title:	Effect of Intrawound Vancomycin Powder in Operatively Treated High Risk Tibia Fractures
Objective:	To examine the effect of intrawound vancomycin powder in reducing deep SSI
Setting:	36 trauma centres across the United States
Level of evidence:	High
Design:	Randomized controlled trial
Selection of participants:	Randomized
Follow-up interval:	12 months
Control group:	Systemic antibiotics
Intervention group:	Vancomycin powder
Primary outcome:	Deep SSI within 182 days
Definition of infection:	Modified CDC criteria for deep SSI
Qadir et al. (2021) [7]	
Title:	Vancomycin Powder Use in Fractures at High Risk of Surgical Site Infection
Objective:	To determine if vancomycin powder reduces infection in high-risk open fractures
Setting:	University of Maryland Shock Trauma Centre, United States
Level of evidence:	Low
Design:	Retrospective cohort
Selection of participants:	Consecutive
Follow-up interval:	6 months
Control group:	Systemic antibiotics
Intervention group:	Vancomycin powder
Primary outcome:	Deep SSI
Definition of infection:	Culture positive infection, return to theatre
Vaida et al. (2020) [8]	
Title:	Evaluating the Efficacy of Topical Vancomycin Powder in Open Lower Extremity Fractures
Objective:	To assess the effect of vancomycin powder on infection in open lower limb fractures
Setting:	West Virginia University, United States
Level of evidence:	Low
Design:	Retrospective cohort
Selection of participants:	Historical vs. intervention group
Follow-up interval:	2 months
Control group:	Systemic antibiotics
Intervention group:	Vancomycin powder
Primary outcome:	Infection, wound complications
Definition of infection:	Confirmed by intraoperative cultures and return to theatre
Pesante and Parry (2024) [9]	
Title:	The Effect of Vancomycin and Tobramycin Local Antibiotic Powder on Surgical Site Infections After Open Treatment of Fracture: A Retrospective Propensity-Matched Analysis
Objective:	To identify the effect of vancomycin and tobramycin powder/PMMA beads intra-operatively
Setting:	University of Louisville, United States
Level of evidence:	Low
Design:	Retrospective cohort
Selection of participants:	Consecutive
Follow-up interval:	Control; 20.9 months. Intervention: 17.5 months
Control group:	Systemic antibiotics
Intervention group:	Vancomycin and tobramycin powder/PMMA beads
Primary outcome:	Wound infection and/or bone infection
Definition of infection:	Clinical diagnosis and/or need for reoperation
Taylor et al. (2024) [10]	
Title:	Effect of Topical Antibiotic Powder in ED on Deep Fracture Related Infections in Type III Open Fractures
Objective:	Assess if ED application of antibiotic powder reduces FRI
Setting:	University of South Florida, United States
Level of evidence:	Low
Design:	Prospective cohort vs. historical control
Selection of participants:	All patients with type III open lower extremity fractures
Follow-up interval:	6 months
Control group:	Systemic antibiotics
Intervention group:	Vancomycin and tobramycin powder
Primary outcome:	Deep FRI
Definition of infection:	Clinical FRI definition with 6-month follow-up

Overview of the Selected Studies

SSI was the primary outcome in all included studies. Although the definition of SSI varied slightly between studies, all adhered to recognized clinical standards. Balabanova et al. classified deep SSIs as those requiring operative debridement [5]. Similarly, O'Toole et al. employed a modified version of the CDC National Healthcare Safety Network Criteria, identifying only deep SSIs that required surgical intervention within 182 days of definitive fixation [6]. Similarly, Qadir et al. followed the CDC definition, including infections located deep to the subcutaneous layer that necessitated operative debridement [7]. Vaida et al. defined infection as any postoperative complication requiring return to the operating theatre, with confirmation via intraoperative cultures [8]. Pesante and Parry applied the fracture-related infection (FRI) consensus definition, requiring debridement with criteria such as purulence, sinus tract formation, or positive microbiological findings [9].Likewise, Taylor et al. used the FRI definition, identifying infections based on clinical assessment and postoperative follow-up within a six-month period [10].

All six included studies reported the intrawound use of topical antibiotics with varying combinations and timing of application. Vaida et al., O'Toole et al., and Qadir et al. utilized vancomycin powder alone, administered intraoperatively within the first operation [6-8]. However, Vaida et al. and O'Toole et al. applied the powder directly into the surgical wound, while Qadir et al. reported application during wound closure [6-8]. Balabanova et al. also applied the antibiotics intraoperatively during closure but used a combination of vancomycin and tobramycin powders [5]. Pesante and Parry employed a similar combination, that is, vancomycin and tobramycin in powder or putty form, placed directly into the surgical wound during the procedure [9]. Notably, Taylor et al. was the only study to apply the antibiotics preoperatively in the emergency department, using vancomycin and tobramycin powder administered directly into the open fracture site prior to formal surgical intervention [10].

The quality of the evidence ranged from low to moderate. The quality of the single RCT included was rated moderate because of missing data and potential selective reporting despite a strong design. In the other cohorts, Vaida et al. and Balabanova et al. were assessed as having low-quality evidence due to retrospective designs, limited adjustment for confounders, and unclear protocol adherence [5,8]. Taylor et al. and Qadir et al. showed moderate quality of evidence, with efforts at matching and consistent outcome definitions, but potential issues remained due to the use of historical controls, some follow-up loss, and intervention variability [8,10]. From all cohorts, Pesante and Parry demonstrated having a better quality of evidence than others, as it supported the propensity score matching, consistent intervention application, and well-defined outcomes; however, it was surgeon-based patient selection, which may have affected generalizability [9].

The effect of topical antibiotic combined with systemic antibiotic prophylaxis versus systemic antibiotic prophylaxis on SSI and fracture site infection was analyzed for every individual study. The overall infection rate in the control groups across these studies ranged from 8.3% to 17.1%. In the treatment groups, vancomycin only ranged from 0% to 8.7%, while vancomycin and tobramycin ranged between 7.7% and 13%.

Meta-Analysis Results

A total of 2224 patients received IV antibiotics alone, while 758 patients received both IV and topical antibiotics. SSI occurred in 10.12% of patients treated with IV-only therapy versus 7.26% in the combined therapy group.

Using a Z-test for two independent proportions, the absolute risk reduction was 2.86%, with a p-value of 0.0197 and a 95% confidence interval ranging from 0.46% to 5.27%. The difference was statistically significant, suggesting a benefit with adjunctive topical antibiotics.

**Figure 2:**
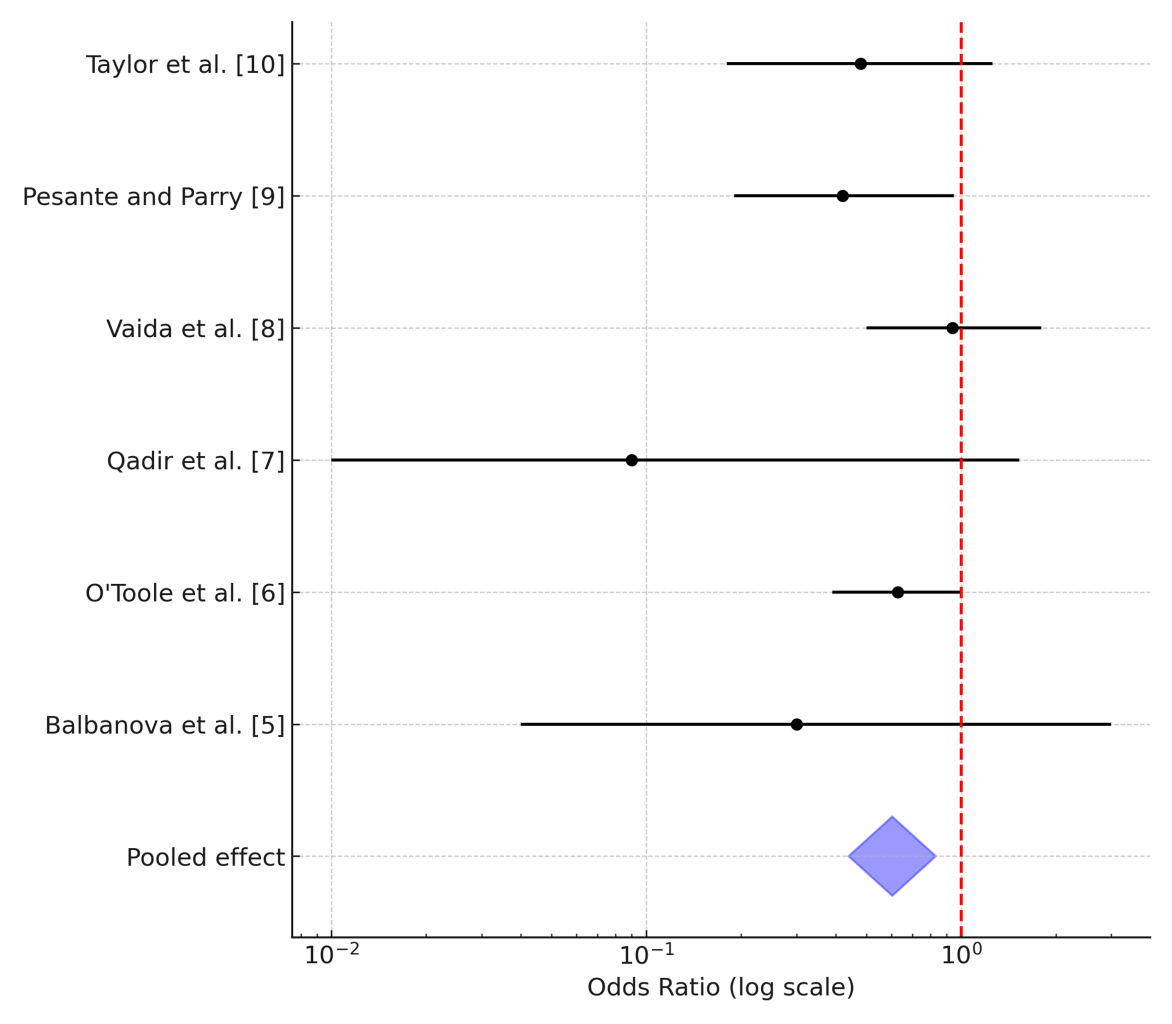
Surgical site infection systemic vs. combined treatment in forest plot

The forest plot (Figure 2) has been drawn using standard convention, including heterogeneity statistics of I^2^, indicating moderate heterogeneity of 42% and Tau^2^ of 0.0134.

Monotherapy vs. Dual Topical Therapy

Among the 758 patients who received topical antibiotics, 562 were treated with vancomycin alone, and 196 received a combination of vancomycin and tobramycin.

The SSI rate was 6.23% in the vancomycin-only group and 10.20% in the dual therapy group. Although the infection rate appeared higher in the dual therapy group, the difference did not reach statistical significance (p=0.0602). Confidence intervals for both groups overlapped, and the observed trend may reflect confounding by indication: dual therapy was likely used in more severe or contaminated injuries. This introduces clinical heterogeneity that limits the interpretation of pooled results.

Secondary Outcomes

Data regarding secondary outcomes such as antibiotic resistance emergence, length of hospital stay, reoperation rates, and cost-effectiveness were inconsistently reported and therefore could not be meta-analyzed. These outcomes are discussed narratively in the Discussion and Limitations section, and their absence is identified as a key limitation of the current evidence base.

Assessment of Publication Bias

**Figure 3:**
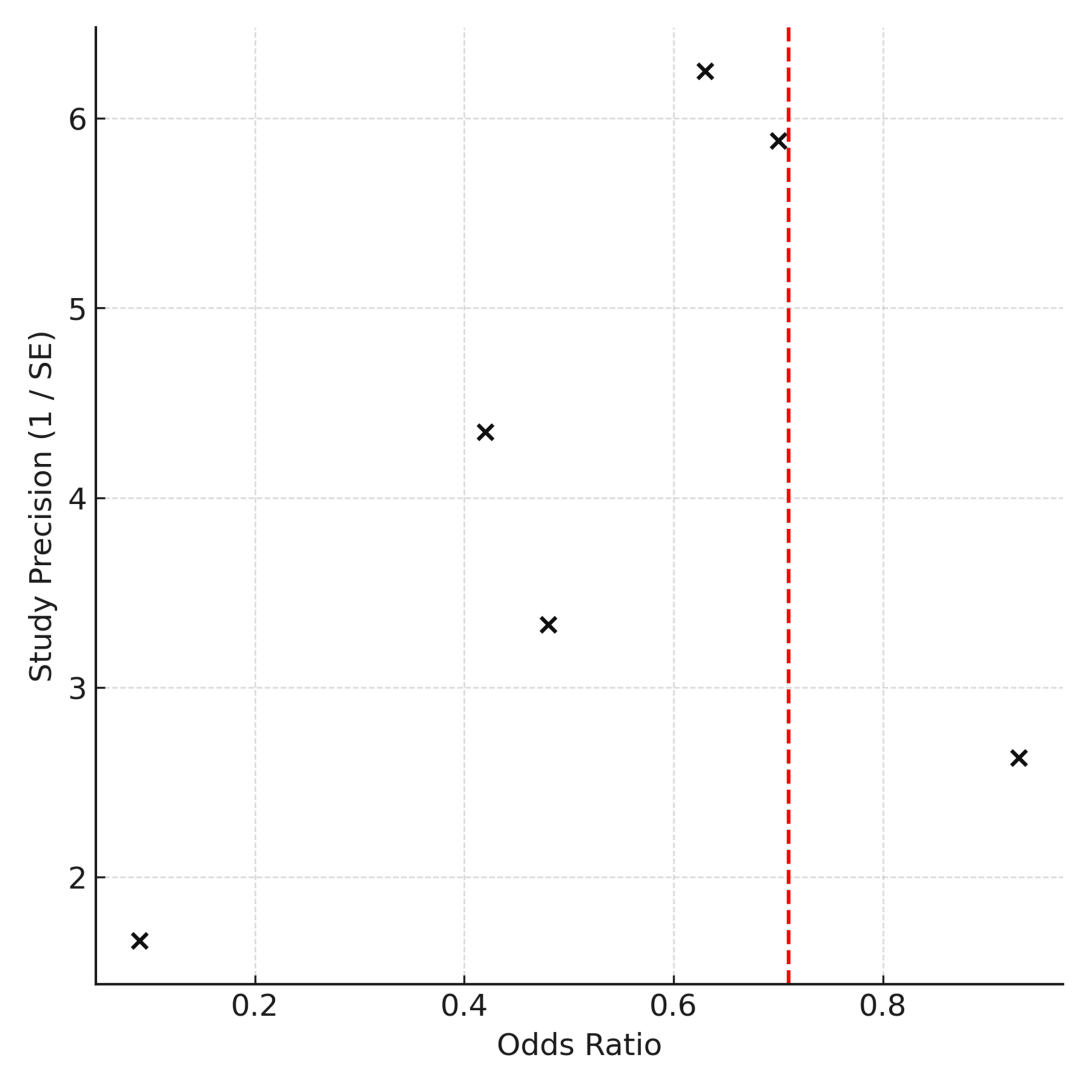
Funnel plot

A funnel plot (Figure 3) was generated to assess publication bias among the six included studies. Due to the small number of studies, Egger's regression test was not conducted, as its statistical power is limited when fewer than 10 studies are included. Visual inspection of the funnel plot did not reveal strong asymmetry, though minor bias cannot be excluded.

Discussion

SSI in the context of open fractures remains a significant cause of morbidity, prolonged hospitalization, and healthcare expenditure [11]. Despite early systemic antibiotic administration and timely operative management, particularly in severe cases such as Gustilo-Anderson type III fractures, infection rates remain unacceptably high [11]. In recent years, the application of topical antibiotics has gained interest as a potential adjunct to systemic antibiotic prophylaxis, with the aim of further reducing SSI in high-risk injuries.

This systematic review identified one RCT and five cohort studies evaluating the prophylactic role of topical antibiotics to reduce SSIs in open fractures. The RCT by O'Toole et al. found a lower rate of deep infection with the use of intrawound vancomycin powder (6.4% vs. 9.8%), though this difference did not reach statistical significance (p=0.06; OR: 0.63; 95% CI 0.39-1.01) [6]. However, a post hoc analysis demonstrated a statistically significant reduction in gram-positive infection (p=0.02), suggesting a potential efficacy of vancomycin against common wound contaminants such as *Staphylococcus aureus* [6].

In line with this, Qadir et al. retrospectively reported no infections in patients treated with intraoperative vancomycin compared to a 10.6% infection rate in the control group (p=0.04; OR: 0.09; 95% CI 0.01-1.53) [7]. Although the sample size of the intervention group was limited (n=35), the results are encouraging and highlight the potential role of high topical antibiotic concentration in disrupting bacterial biofilm, especially crucial in the context of compromised soft tissue often seen in open fractures [7].

Nevertheless, concerns about the limited spectrum of single-agent vancomycin therapy were raised in the O'Toole et al. trial, which observed persistent infections due to gram-negative organisms [6]. Addressing this, Pesante and Parry investigated the combined use of tobramycin and vancomycin powders in patients with open fractures, reporting a significant reduction in deep SSIs (4.6% vs. 13%; p=0.04; OR: 0.42; 95% CI: 0.19-0.95) [9].

This supports the concept that broad-spectrum topical antibiotic coverage may be more effective, particularly in polymicrobial infections commonly associated with high-grade open fractures. The ongoing TOBRA RCT (NCT05849090) aims to provide high-quality evidence on whether the addition of tobramycin to vancomycin gives further benefit in preventing SSIs in open fractures [12].

The findings from this comparative analysis, done prior to it, provided compelling statistical evidence supporting the role of topical antibiotic administration as an adjunct to intravenous therapy in reducing SSI. Firstly, although no statistically significant difference was observed between the use of vancomycin alone and the combination of vancomycin with tobramycin, the combination therapy demonstrated a numerically lower infection rate, warranting further investigation in larger, controlled cohorts. More notably, the comparison between intravenous-only treatment and combined intravenous with topical antibiotic administration revealed a statistically significant reduction in SSI incidence (p=0.0197), with an absolute infection rate difference of 2.86%. The 95% confidence interval (0.46% to 5.27%) affirms the robustness of this finding and suggests a clinically meaningful benefit. These data collectively support the adjunctive use of topical antibiotics as an effective strategy to enhance infection prophylaxis in SSI. Further high-powered, prospective studies are recommended to confirm these findings and optimize antimicrobial regimens for orthopaedic trauma care.

Similarly, Taylor et al. evaluated the use of combined antibiotic powder in the emergency department, reporting a reduction in infection rate from 17.05% to 9.09% [10]. Burbank et al. also demonstrated that early application of topical antibiotic powder in the emergency setting was associated with a reduced bacterial burden and lower risk of SSI, highlighting the importance of timely intervention before definitive fixation [13]. Wheeler et al. further reported that the placement of antibiotic powder within 24 hours of injury significantly reduced infection rates, supporting the concept of very-early prophylaxis in open fractures [14]. Although Taylor et al. did not demonstrate statistical significance (p=0.133; OR: 0.48; 95% CI: 0.18-1.26), the study introduced an important consideration: early topical prophylaxis at the time of presentation may help reduce bacterial burden prior to definitive surgical intervention [10]. 

However, not all studies demonstrated benefit. In the study by Vaida et al., there was no significant difference in infection rates with vancomycin powder use (8.7% vs. 9.28%; p=0.901) [8]. Moreover, the study highlighted a potential downside, showing a significantly higher rate of wound healing complications in the vancomycin group (15.2% vs. 6.4%; p=0.039) [8].

This finding remained significant even when controlling for confounding variables such as fracture grade, injury severity score, comorbidities, and demographic factors [8]. ​​​The reported complications included wound dehiscence, unplanned application of negative pressure wound therapy, debridement, chronic wound formation, and skin necrosis [8]. Additionally, previous studies have associated topical vancomycin use with sterile seroma formation [15,16].​​Seroma formation may result from the osmotic properties of the powder, which can draw fluid into the wound bed, particularly when placed in large amounts or in closed spaces [17]. This accumulation of sterile fluid may mimic infection, delay wound healing, and lead to unnecessary intervention [8]. However, it is worth noting that in the RCT by O'Toole et al., there was no statistically significant difference in wound dehiscence (p=0.42) or non-union rates (p=0.43) between the intervention and control groups [6].

Safety and systemic complications were also explored. Balabanova et al. reviewed the complications associated with topical antibiotics, finding no increased incidence of systemic adverse effects (2% vs. 5%) [5]. The study argued that serum levels of topically applied antibiotics seldom reach supratherapeutic concentrations, thereby reducing the likelihood of systemic toxicity [14]. While acute kidney injury occurred less frequently in the topical group, the difference was not statistically significant (p=0.3; OR: 0.3; 95% CI: 0.04-3) [5].Similarly, O'Toole et al. reported no increase in systemic complications with topical antibiotic use [6]. Stauss et al. confirmed that the intra-articular administration of vancomycin powder results in negligible systemic absorption, further supporting its safety profile with respect to nephrotoxicity and other systemic complications [18].

A recurring concern among the studies reviewed is the potential for the development of antibiotic resistance. Balabanova et al. referenced CDC caution regarding routine topical antibiotic use in surgical wounds due to the theoretical risk of promoting antimicrobial resistance [5]. However, to date, no robust clinical evidence has definitively linked topical antibiotic use in open fractures to higher rates of antimicrobial resistance. Nonetheless, this highlights the need for future research into microbial surveillance and long-term ecological consequences to topical antibiotic strategies.

Notably, none of the included studies directly assessed hospital length of stay as a primary or secondary outcome. Given that infected fractures substantially increase hospital costs and length of stay, as highlighted by O'Connor et al., incorporating these economic outcomes into future trials would provide a more comprehensive assessment of cost-effectiveness [17], and this represents an important gap in the literature.

Future studies should consider hospital length of stay as an important economic and clinical endpoint to fully assess the cost-effectiveness of prophylactic topical antibiotics in open fractures. It is worth noting that reoperation rates and numbers were not reported in the aforementioned studies. This represents a significant limitation, as such data would be essential to determine whether the use of topical antibiotics contributes to a reduction in the need for reoperations. Additionally, the current literature does not explore alternative forms of topical antibiotic application. Further research is recommended to evaluate the potential role of these alternative formulations in the management of open fractures.

From a cost-effectiveness point of view, vancomycin powder is not expensive and easy to administer, making it an attractive option, especially in a resource-limited environment [19].​​​​​ However, the lack of standardized protocol and inconsistent evidence of topical antibiotics highlights the need for further high-quality research.

Also, while no overt asymmetry was observed in the funnel plot, the small number of studies limits confidence in this assessment. The potential for publication bias remains, particularly given the absence of grey literature, negative studies, or trial registries. A recent meta-analysis by Saka et al. found that topical vancomycin reduced infection rates in some high-risk orthopaedic procedures but overall evidence remains inconclusive, reinforcing the need for further randomized trials [20]. Future meta-analyses should aim to include unpublished data and trial protocols where possible. 

Recommendations for Future Research

To strengthen the evidence base, future research should prioritize large, multicentre RCTs directly comparing adjunctive topical and systemic therapy with systemic therapy alone. Standardized definitions of SSI and wound complications are essential to improve comparability across studies. Stratification by fracture severity, contamination level, and timing of antibiotic application would provide more nuanced insights into effectiveness. Incorporating microbial surveillance is important to monitor the potential development of antibiotic resistance. Furthermore, health economic evaluations should assess cost per infection avoided, length of hospital stay, and reoperation rates. Finally, long-term safety data are needed to evaluate outcomes related to wound healing, non-union, and systemic effects.

Strengths and Limitations

This systematic review included the current evidence on the use of topical antibiotics in open fractures, comprising one RCT and five cohort studies with a combined sample size of nearly 3000 patients. Strengths include adherence to PRISMA 2020 methodology and a clearly defined PICOS framework. Cohort studies employed propensity matching techniques and retrospective cohort designs with large control groups to enhance internal validity. Consistent protocols were used throughout, with uniform intravenous antibiotic regimens and surgical techniques aimed at minimizing confounding variables.

However, the review has several limitations. Most of the studies were retrospective and conducted in the United States, limiting the generalizability of findings to other healthcare settings, particularly in low- and middle-income countries. Variability in topical antibiotic type, dosage, application timing, and definition of infection introduced clinical heterogeneity, reflected by a moderate I^2 ^of 42%. Most studies lacked adjustment for key confounders such as contamination level and comorbidities.

Importantly, several relevant outcomes, including reoperation rates, length of hospital stay, and microbial resistance surveillance, were not reported or inconsistently presented.

## Conclusions

Topical antibiotic use, when combined with systemic prophylaxis, appears to reduce SSI rates in open fractures. The pooled analysis demonstrated a statistically and clinically significant reduction in SSI, particularly in high-risk injuries. Importantly, topical antibiotics were not associated with an increase in systemic adverse events, although one study reported a higher incidence of local wound complications. While this suggests that topical antibiotics can be considered relatively safe, their application should be guided by careful patient selection and clinical judgment, given the variability in evidence quality.

Despite encouraging findings, caution is warranted due to heterogeneity in study designs and the predominance of retrospective data. Significant gaps remain, including the absence of microbial resistance monitoring, cost-effectiveness analyses, and long-term safety outcomes such as fracture healing and non-union. Future high-quality, multicentre RCTs are required to address these limitations, employing consistent outcome definitions, incorporating microbial surveillance, and ensuring applicability across diverse healthcare settings. Until such evidence emerges, topical antibiotics should be regarded as a promising adjunct rather than a definitive standard of care.

## Appendices

Full search strategy

*Databases Searched* • PubMed • Cochrane Library (CENTRAL) • Scopus

*Search Date* • February 2025

*Search Limits* • Publication years: 2015 to 2025 • Language: English • Human studies only

1. PubMed Search Strategy

("Open Fractures"[Mesh] OR "open fracture*" OR "compound fracture*") AND ("Anti-Bacterial Agents"[Mesh] OR "topical antibiotic*" OR "local antibiotic*" OR "intrawound antibiotic*" OR "vancomycin powder" OR "tobramycin powder") AND ("Surgical Wound Infection"[Mesh] OR "surgical site infection*" OR "SSI" OR "fracture-related infection") AND ("Trauma"[Mesh] OR "orthopaedic trauma") Filters applied: English, 2015 to 2025

2. Cochrane Library Search Strategy

(open fracture* OR compound fracture*) AND (topical antibiotic* OR local antibiotic* OR vancomycin powder OR tobramycin powder) AND (surgical site infection* OR SSI OR wound infection OR fracture-related infection) AND (trauma OR orthopaedic trauma) Publication year: 2015 to 2025

3. Scopus Search Strategy

TITLE-ABS-KEY("open fracture*" OR "compound fracture*") AND TITLE-ABS-KEY("topical antibiotic*" OR "local antibiotic*" OR "vancomycin powder" OR "tobramycin powder") AND TITLE-ABS-KEY("surgical site infection" OR "SSI" OR "fracture-related infection") AND TITLE-ABS-KEY("trauma" OR "orthopaedic trauma") LIMIT-TO(PUBYEAR, 2015-2025) AND LIMIT-TO(LANGUAGE, "English")
